# Larval Food Limitation in a *Speyeria* Butterfly (Nymphalidae): How Many Butterflies Can Be Supported?

**DOI:** 10.3390/insects9040179

**Published:** 2018-12-02

**Authors:** Ryan I. Hill, Cassidi E. Rush, John Mayberry

**Affiliations:** 1 Department of Biological Sciences, University of the Pacific, 3601 Pacific Ave, Stockton, CA 95211, USA; c_rush1@u.pacific.edu; 2 Department of Mathematics, University of the Pacific, 3601 Pacific Ave, Stockton, CA 95211, USA; jmayberry@pacific.edu

**Keywords:** *Viola*, *Viola purpurea*, *Speyeria adiaste*, bottom–up, top–down, insect conservation

## Abstract

For herbivorous insects the importance of larval food plants is obvious, yet the role of host abundance and density in conservation are relatively understudied. Populations of *Speyeria* butterflies across North America have declined and *Speyeria adiaste* is an imperiled species endemic to the southern California Coast Ranges. In this paper, we study the link between the food plant *Viola purpurea quercetorum* and abundance of its herbivore *Speyeria adiaste clemencei* to better understand the butterfly’s decline and aid in restoration of this and other *Speyeria* species. To assess the degree to which the larval food plant limits adult abundance of *S. a. clemencei* in 2013, we compared adult population counts to population size predicted from a Monte Carlo simulation using data for number of *V. pur. quercetorum* plants, number of leaves per plant, and leaf area per plant, with lab estimates of leaf area consumed to reach pupal stage on the non-native host *V. papilionacea*. Results indicated an average estimate of 765 pupae (median = 478), with 77% of the distribution being <1000 pupae. However, this was heavily dependent on plant distribution, and accounting for the number of transect segments with sufficient host to support a pupa predicted 371 pupae. The adult population empirical estimate was 227 individuals (95% CI is 146 to 392), which lies near the first quartile of the simulated distribution. These results indicate that the amount of host *available* to larvae was more closely linked to adult abundance than the amount of host *present*, especially when considering assumptions of the analyses. The data also indicate that robust populations require host density well in excess of what is eaten by larvae, in combination with appropriate spacing, to mitigate factors such as competition, starvation from leaving host patches, or unrelated to food plant, such as mortality from drought, predators, parasites, or disease.

## 1. Introduction

Interactions among plants, herbivores and parasitoids/predators have received considerable attention in the ecological literature. Insect herbivores have played a prominent role in these studies to answer questions ranging from “why is the world green?” to “what is the relative role of bottom–up and top–down effects in controlling herbivore abundance?” to “what factors modulate resource limitation and predation in a system?” [1,2,3,4,5]. These classic ecological questions have been applied to conservation biology less often, yet they clearly relate to issues of population decline, restoration, and management of plants and herbivores [6,7,8,9]. For example, are species declining because of bottom–up resource limitation, or changes to top–down effects of predators or parasites, or an interaction between the two?

Studies of butterflies across the globe have documented marked decreases in populations [10,11,12,13,14,15,16,17]. A powerful example of this in North America is seen in the decline of populations of *Speyeria* butterflies along with their native *Viola* larval host plants [18]. *Speyeria* populations across North America have been affected, from the relatively large sized eastern North America species *Speyeria idalia* [8], and *Speyeria diana* [19,20], with their once large geographic distributions, to *S. nokomis* in the southwestern U.S. [18], to smaller sized species with many described geographic subspecies in western North America such as *S. hydaspe* [18], *S. zerene* [18,21], *S. egleis* [22], *S. adiaste* [23,24] and *S. callippe* [25]. Each of these species has reportedly declined in abundance or number of populations. Some of the taxa of western species have received federal listing as endangered (e.g., *S. zerene behrensii*, *S. zerene myrtleae* and *S. callippe callippe*), or threatened (e.g., *S. zerene hippolyta*), but the rest have not.

Reasons for decline of *Speyeria* butterflies center mainly on human habitat alteration and associated effects on their *Viola* larval food plants. *Speyeria* and *Viola* are very sensitive species, and human disturbances have been implicated in altering habitats to be less suitable for *Viola* food plants by cutting and thinning forests, overgrazing or water diversion ([18], RIH personal observation), or destroying habitats altogether via development, agriculture, and natural resource extraction such as mining ([18], RIH personal observation). Alteration of habitats resulting from fire suppression or prescribed fire, or overgrowth of non-native plants such as European grasses in California, have also been discussed [8,22,26]. Fire and grazing may have positive or negative effects in different systems depending on their intensity. For example, *S. egleis egleis* have been documented to colonize new areas after wild fire, with declines associated with succession and overgrowth afterward [22], and *S. idalia* larvae appear capable of surviving low to moderate surface fires [27]. For *S. diana*, prescribed fire appears to help with adult nectar sources [28], but repeated fire may harm *Viola* populations [8]. Overgrazing is detrimental to *Viola* [18], but the correct amount of grazing can be beneficial for managing grasslands by slowing or stopping succession and reducing non-native grasses that overgrow native plants such as *Viola* spp. In the California Coast Ranges many grazed grasslands show the most robust *S. callippe* and *S. coronis* populations (RIH personal observation). Climate change and drought are also implicated in declines of *Speyeria* populations [20,22,29] and presumably their *Viola* hosts.

*Speyeria* populations clearly need adequate *Viola* populations, but what adequate is, and many other aspects of *Speyeria* larval ecology have only recently been investigated or are not well understood. For example, Kelly and Debinski [8] demonstrated that larger populations of *S. idalia* were significantly correlated with larger habitat areas, and by extension access to more *Viola* food plants. However, adult *S. idalia* population size was not significantly correlated with *Viola* host density in their analysis, leaving unresolved the question of whether the food plant was limiting [8]. Data on biomass consumption of *Speyeria* larvae are still lacking, and as suggested by Kelly and Debinski [8], data on the biomass required by a larva, and an entire population, as well as how food limitation relates to fecundity, would be of conservation value. Other recent work has demonstrated that *Speyeria zerene* larvae did not locate their food plants well and only find them when they are very close (~3 cm) [30], and that host density is very important in modeling larval survival in this species [31]. Thus although it is clear that large amounts of dense *Viola* host will provide the strongest populations, the link between larval host abundance and adult abundance has rarely been investigated [8], and few data exist on field estimates of host abundance and density, leaving open the question of how much larval food plant is required for a given population size, and what the threshold abundance is to support a population [8]. Therefore, identifying the amount of *Viola* necessary to sustain a given *Speyeria* larval population until adult form is important for better understanding *Speyeria* larval ecology.

Here we focus on a particular species of conservation concern in southern California, *Speyeria adiaste* (W.H. Edwards 1864), to continue investigations of its autecology and answer to what degree the food plant is limiting. *S. adiaste* is endemic to the southern California Coast Ranges and Transverse ranges [32,33] and is currently without threatened or endangered status. Within *S. adiaste* are three described subspecies, which from north to south are *S. a. adiaste*, *S. a. clemencei*, and *S. a. atossa*. *Speyeria adiaste atossa* (Santa Barbara, Ventura, Los Angeles, and Kern Cos.) is considered extinct because it has not been seen since 1960 [22], making *S. adiaste* the only *Speyeria* species to have a described subspecies go extinct (but see [34] for discussion of *S. zerene*). Of the remaining two subspecies, *Speyeria adiaste clemencei* (Monterey to San Louis Obispo Co.) is considered the most abundant, although the number of widely spaced remaining populations, and their sizes, remains unknown (but see [24]). *Speyeria adiaste adiaste* (San Mateo Co., Santa Cruz Co. and Santa Clara Co.) has become increasingly rare and suffered population declines. *Speyeria adiaste* has been petitioned for endangered status because of declining populations, but was rejected based on a lack of available information concerning the species. Recent work on *S. a. clemencei* documented population sizes of several hundred individuals on Chews Ridge at best, followed by marked declines during the study period [24]. This potential for drastic changes in population size coupled with the seemingly widely spaced populations of *S. adiaste* suggests this species exists as a metapopulation and will require adequate populations of *Viola* hosts to colonize and maintain viable subpopulations. However it is unknown what these *Viola* populations should look like. Thus, goals of this project are to provide baseline data on *Viola* food plant population abundance and density, and explore the link between food plant and *Speyeria adiaste* abundance. This information can elucidate how many adult butterflies a specific habitat should theoretically support and can be an important component guiding how *Speyeria* and *Viola* habitats should be managed and restored.

## 2. Materials and Methods

### 2.1. Study Site

**Figure 1:**
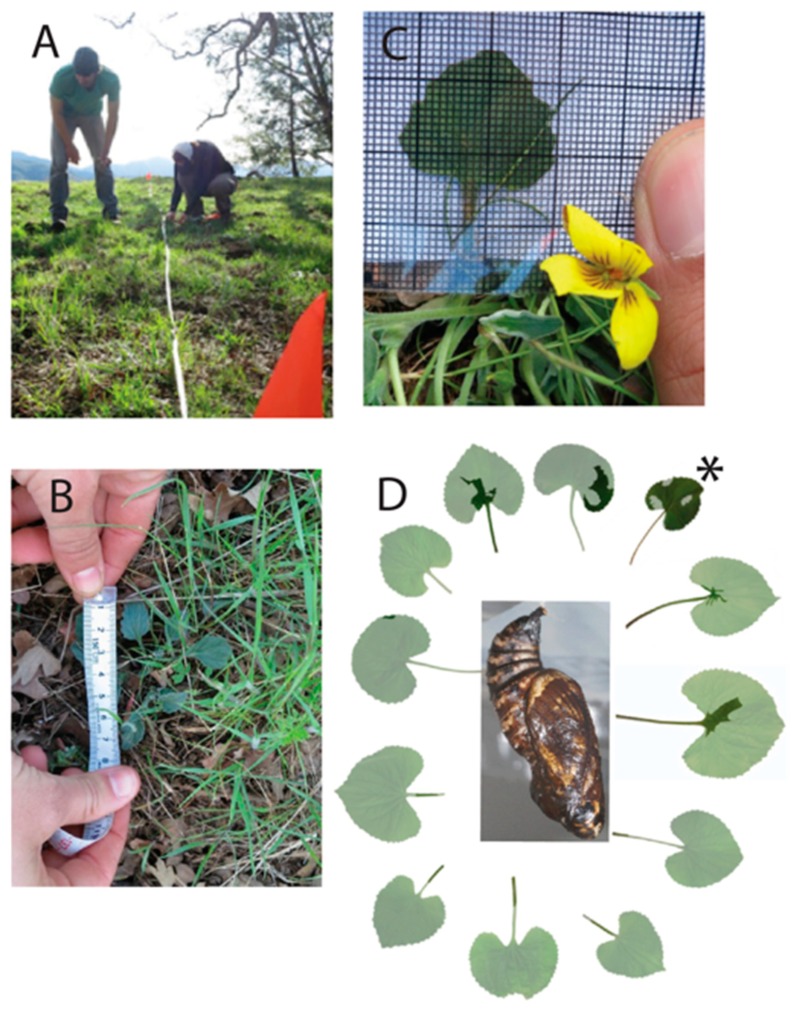
Estimating area of *Viola purpurea quercetorum* and area of consumed *V. papilionacea*. (**A**) Abundance transect on Chews Ridge. (**B**) Assessing coverage and number of leaves per plant. (**C**) Quantifying leaf area per leaf. (**D**) Consumption of rearing host *Viola papilionacea*, pale leaf shows plant before consumption and dark overlay shows remaining leaf after consumption. Asterisk indicates first leaf consumed, with subsequent leaves arranged clockwise.

Field portions of this study took place on Chews Ridge in Monterey Co., California (36.31336°; −121.57323°, 1500 m elevation) in 2013. This locality has a robust, but declining population of *S. adiaste clemencei* [23,24], and fieldwork was conducted in the same 243,206.5 m^2^ area as in Zaman, Tenney, Rush and Hill ([24]; see Figure 1). The study area contains slopes of mixed oak-pine woodland having areas of relatively open yellow-pine and oak woods alternating with moist to dry meadows.

### 2.2. Abundance of Adult Butterflies and Food Plant

The larval food plant of *S. adiaste clemencei* at Chews Ridge is *Viola purpurea* Kellogg subsp. *quercetorum* (M.S. Baker & J.C. Clausen) R.J. Little [23]. We conducted transect counts to estimate the amount of available *Viola purpurea quercetorum* leaf area on Chews Ridge. On each transect the number of individual plants was counted, and these data were combined with data on the number of leaves per plant, the leaf area, and the total area of the study site.

Parallel belt transects (*n* = 135) were made every 16 m and extended from the lower study area boundary to the ridge crest. In total, 12,442 m of linear distance was sampled, with an average transect length of 92.16 m. A compass was used to orient each transect to 40° relative to true North to ensure transects were parallel. Each transect was broken into segments of 30 m maximum length (*n* = 475 segments overall, *n* = 371 of 30 m), and GPS data were recorded at the start and end of each segment. Approximately 270 person hours over 9 days of fieldwork were completed. *Viola pur. quercetorum* within one meter of each side of the transect line were counted (Figure 1A), giving a maximum sampling area of 60 m^2^ per 30 m segment, and a total transect area of 24,883 m^2^ sampled across the study site.

*Viola pur. quercetorum* individuals were relatively easy to distinguish, even when clumped together allowing us to tally the total number of individual violets. However, we also recorded data on cover by measuring the length along the longest dimension and width perpendicular to that, for each violet or patch of violets (Figure 1B). To obtain data for number of leaves per plant, we counted the number of leaves per plant on the first five to 20 plants in haphazardly chosen segments among 28 transects. In total 241 plants were sampled for leaf number per plant. Given that sample size was low for this variable based on data collection in 2013 (*n* = 85), we returned to collect additional data in 2014. There was no difference in mean leaf number per plant between years (equal variance t_239_ = 0.11, *p* = 0.91, test with unequal variance assumption was qualitatively the same). To calculate leaf area, images were taken of up to 10 leaves per plant/patch, with each leaf placed under a metric printed grid (1 mm × 1 mm) placed on a white background (Figure 1C). Leaves on each plant were selected based on compass direction, beginning at due north, and continuing in a clockwise direction until up to 10 leaves were sampled. In total 349 leaves were sampled for leaf area.

The size of the *S. a. clemencei* population on Chews Ridge was estimated in a related paper using mark-release recapture methods in June and July 2013 (see [24] for details) and so we use those estimates here. The adults marked in June–July 2013 would have fed on the violets available during the April–May 2013 transect counts.

### 2.3. Leaf Area Consumption

*Speyeria adiaste clemencei* were reared in the lab to obtain data on the amount of food plant required for a larva to reach the adult stage. A female *Speyeria a. clemencei* from Chews Ridge was placed within one day of field collection into a brown grocery bag with dried leaves of *Viola pedunculata* Torr. & A. Gray and *V. papilionacea* Pursh. Larvae were retrieved from the bags and put into wood blocks for diapause at 4 °C and later reared in plastic cups with cut leaves of *Viola papilionacea* (purchased from Tennessee Wholesale Nursery). See Zaman, Tenney, Brunell, Chen and Hill [23] for further details. Digital photos were taken of each leaf before and after being offered to a feeding larva, with the difference in area representing leaf area consumed. Spirit levels were used to ensure images were taken with the leaf and camera in the same plane, and the leaf was gently overlaid with a transparency sheet printed with a metric grid (1 mm^2^ cells). Cells in the grid with more than half of the area containing leaf were counted. To estimate the amount of leaf area required to become a viable adult, the area consumed was summed across all leaves from first instar to pupation (Figure 1D). We recorded sex and measured forewing length for resulting adults. Forewing length was also measured for field collected males and females. We used an equal variance *t*-test to test the null hypothesis that male and female leaf area consumption was the same (*n* = 4 for each sex, test with unequal variance assumption was qualitatively the same). We used a two-way Analysis of Variance (ANOVA) with interaction to test for differences in forewing length (an estimate of size), between sexes and between lab reared vs. field collected individuals. In total, 10 larvae from the same female were reared on leaves from potted *Viola papilionacea*. This lab host was used because it is a readily available surrogate for wild California *Viola pur. quercetorum*, and no *V. pur. quercetorum* were available. In addition, the morphology of *V. papilionacea* is conducive to image analysis and measurement, *S. adiaste* larval survival was good, and it is more similar in water content to *V. purpurea* than store bought pansies, which can also serve as rearing food plants. Leaf area is an appropriate proxy for amount of food because it correlates strongly with mass [35] and is amenable to quantification before and after larval feeding without as large of an artifact from desiccation, as would be the case with mass.

### 2.4. Model Fitting and Simulation Analysis

To model the distribution of number of leaves per plant, leaf area per leaf, and leaf area consumed we fit the following distributions to each data set using maximum likelihood and the fitdistr() function in R: normal, log-normal, truncated normal, gamma, and Weibull. All but the truncated normal distribution are available as options in R, so we wrote our own code for this distribution (Appendix A). We selected the distribution with the lowest Akaike Information Criterion (AIC) [36] as the best model for each data set and plotted the fitted curves for each distribution to the data to assess the fit. Model fitting results are provided in Appendix B.

Given the large number of segments that lacked *Viola purpurea* individuals, the plant per area data set had many zero values requiring a different model fitting approach. For the plant per area dataset we fit an exponential distribution using maximum likelihood and compared this result to a model that was a mixture between a point mass at zero and an exponential distribution with weights estimated from the proportion of zero values in the data set. A likelihood ratio test was used to test for a significant difference between these nested models.

A Monte Carlo simulation estimating the number of pupae Chews Ridge could potentially support was performed using 10,000 iterations. In each iteration new values were obtained in the following way to model stochastic variation. Data for number of leaves per plant, leaf area per leaf, and leaf area consumed per pupa were obtained by drawing at random from their fitted distributions. Data on number of *V. purpurea* plants per m^2^ was obtained by generating a distribution of 1000 data points based on the fitted mixture model and calculating the resulting mean. This reduced the amount of variation in density in each iteration but was necessary to avoid draws of zero violets. The estimated number of supported pupae in each iteration was calculated with the following formulae:

Leaf Area Present = Area Sampled × *V. purpurea*/m^2^ × #Leaves Per Plant × Leaf Area Per Leaf, (1)

#Pupae = Leaf Area Present ÷ Leaf Area Consumed,(2)

In order to model the effect of available food plant density on the variance in number of potential pupae, an additional simulation analysis was conducted using violet densities ranging from 0.01 to 0.1 plants per m^2^ in 0.001 intervals. This range was chosen to encompass 71% of the empirical data. All modeling and statistics were done using R 3.4.0 [37].

## 3. Results

### 3.1. Viola Purpurea Quercetorum Abundance, Density and Amount of Leaf Present on Chews Ridge

**Figure 2:**
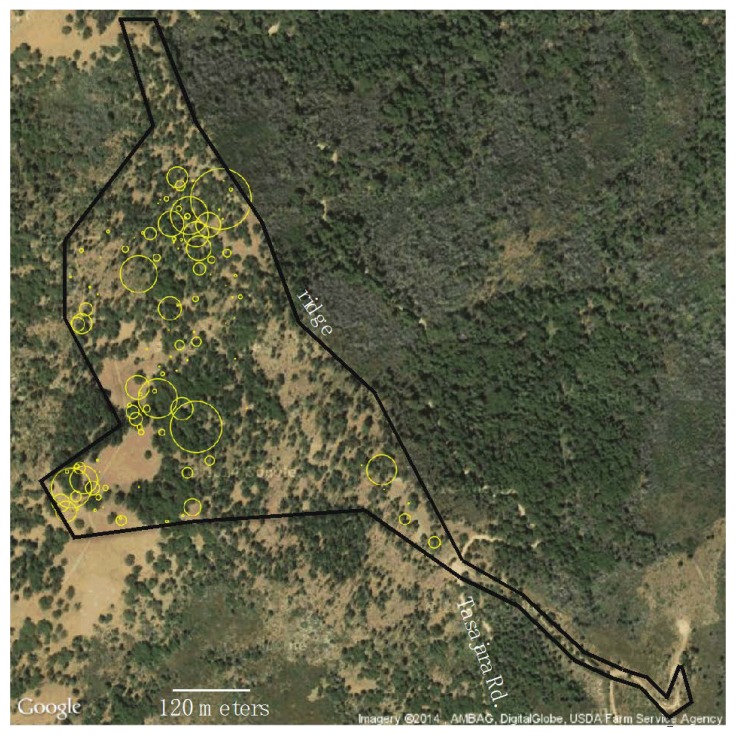
Distribution of *Viola purpurea quercetorum* in the study area. Yellow circles indicate number of *Viola pur. quercetorum* found in each 30 m transect segment. Transects ran diagonally from left to right approximately perpendicular to the ridge.

**Figure 3:**
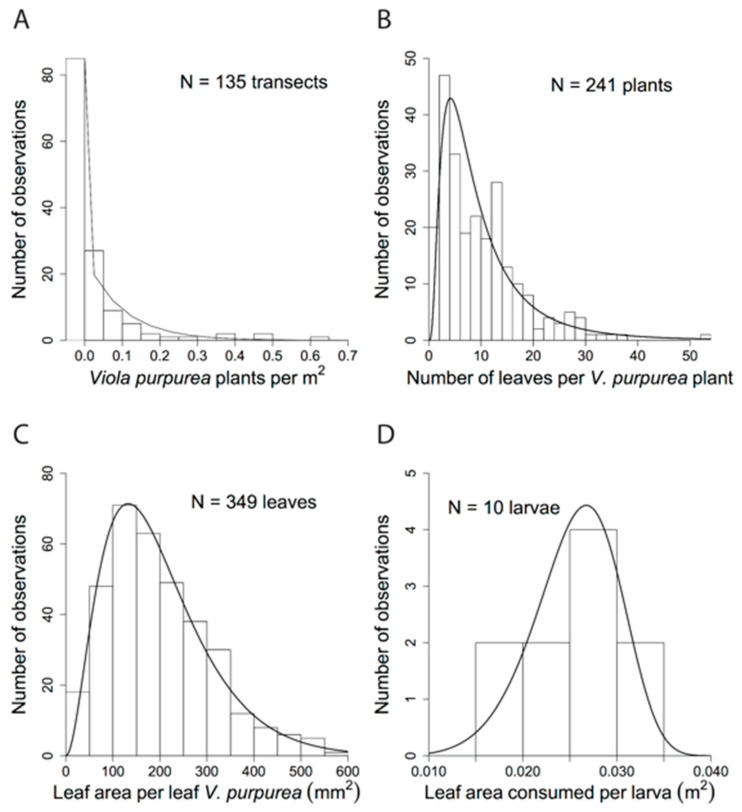
Data histograms and fitted distributions. (**A**) Number of *V. pur. quercetorum* plants per area. (**B**) Number of *V. pur. quercetorum* leaves per plant. (**C**) Variation in leaf area among *V. pur. quercetorum* leaves. (**D**) Leaf area of *V. papilionacea* consumed by *S. adiaste clemencei* larvae.

In total, the transect counts found 1258 *Viola purpurea quercetorum* individuals. The *Viola pur. quercetorum* individuals were not uniformly distributed across the sample area (Figure 2). Instead, they were clumped, and most often found in the more moist woodland and woodland margins in small openings, from relatively flat to more sloped areas, rather than in the more open meadows. Based on the total transect area sampled (24,883 m^2^) a rough calculation of *V. pur. quercetorum* density was 0.051 per m^2^. However, this does not take into account variation between transects (Figure 2), which had a maximum density of 0.65 *Viola* per m^2^ and minimum of 0.0 per m^2^. Therefore a better approach was to estimate the density on each transect and average, which gave an average *Viola* density of 0.037 per m^2^. The mixture model with zero point mass and exponential distribution was a significantly better fit (χ^2^ = 573, *p* < 0.00001) to the *V. pur. quercetorum* per m^2^ data than the exponential distribution. The fit of the mixture model to the data is shown in Figure 3A.

The average number of leaves per *V. pur. quercetorum* plant was 10.3, with a median of 8.5 leaves and standard deviation of 7.9 leaves (Figure 3B). The best fit to the leaf per plant data set was the log-normal distribution (AIC = 1561.5, see Appendix B: Table A1) with parameters meanlog equal to 2.05 and sdlog equal to 0.792.

The average leaf area per leaf of *V. pur. quercetorum* was 198.6 mm^2^ with median 181.0 mm^2^ and standard deviation 111.0 mm^2^ (Figure 3C). The best fit to the leaf area per leaf data was the gamma distribution (AIC = 4219.5, see Appendix B: Table A2) with shape parameter equal to 3.00 and rate parameter equal to 0.0151.

### 3.2. Food Plant Consumption in Lab

All 10 *Speyeria adiaste* reared in the lab pupated to provide data on leaf area consumed to pupation. Two individuals died during the pupal stage and did not eclose; however the leaf area consumed by these individuals was well within the range of the others, and the values suggested they were female. Mean leaf area consumed among all 10 larvae was 253.9 cm^2^ (median = 277.9, s.d. = 53.2 cm^2^) (Figure 3D), and ranged from 163.3 cm^2^ to 310.3 cm^2^. Males and females differed strongly in the leaf area consumed (t_6_ = 5.5, *p* = 0.0015) with females consuming significantly more (mean = 290.6 cm^2^, s.d. = 15.8 cm^2^, *n* = 4) than males (mean = 196.7 cm^2^, s.d. = 30.3 cm^2^, *n* = 4).

The two-way ANOVA testing for effects of lab rearing and sex on forewing length of individuals was significant (F_3,13_ = 10.0, *p* = 0.001). Both sex (F_1,13_ = 16.3, *p* = 0.0014) and lab rearing (F_1,13_ = 13.4, *p* = 0.0029) had significant effects on forewing length. The interaction of sex and lab rearing was not significant (F_1,13_ = 0.27, *p* = 0.61), indicating that forewing length differences were consistent, with males and lab reared individuals being smaller. For wild females average forewing length = 31.56 mm (*n* = 3), versus an average of 28.75 mm (*n* = 4) for lab reared females. For wild males average forewing length = 28.91 mm (*n* = 6), versus an average of 26.71 mm (*n* = 4) in the lab.

The best fit for the leaf area consumption data used to draw from in the simulation analysis was the Weibull distribution (AIC = −74.6; see Appendix B: Table A3), with shape parameter equal to 6.53 and scale parameter equal to 0.027 (Figure 3D).

### 3.3. Simulation and Comparison of Estimated Number Pupae to Adult Population Size

**Figure 4:**
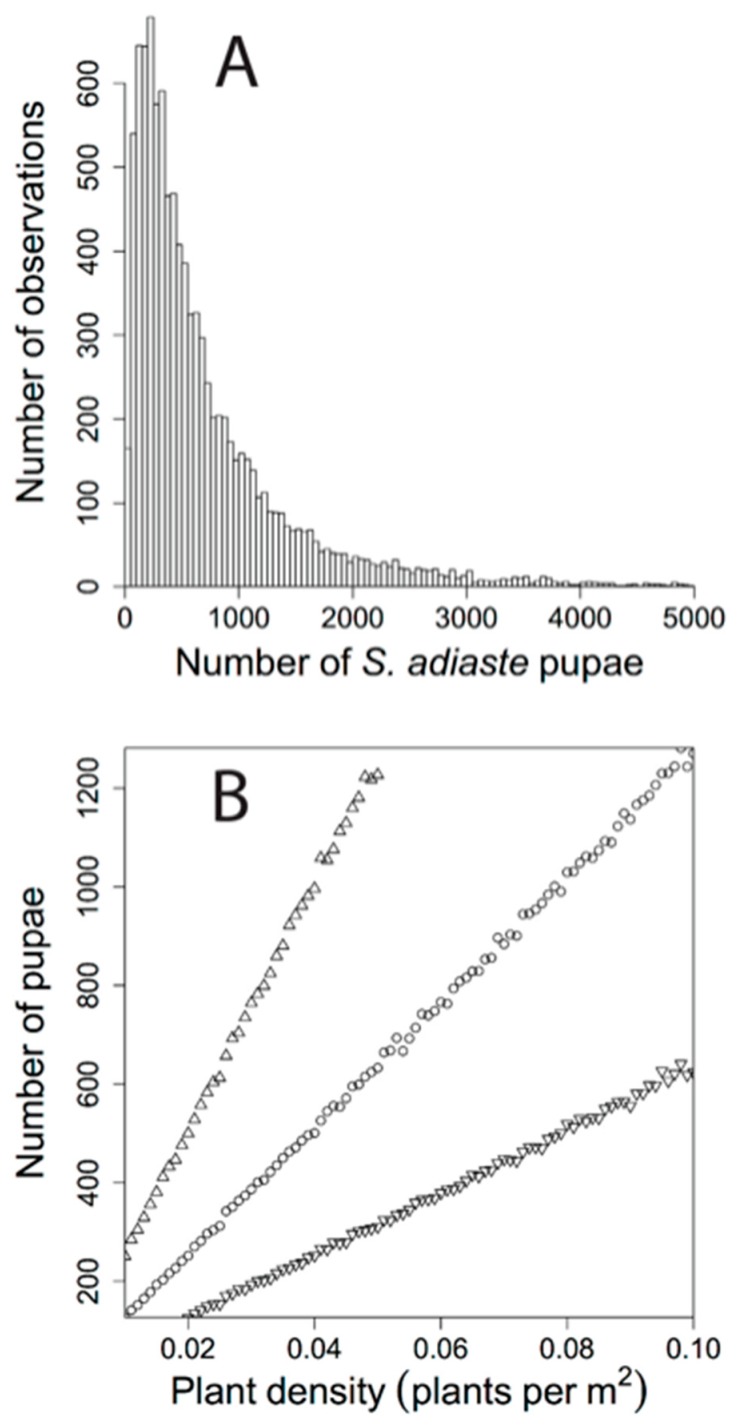
Number of pupae predicted based on simulations drawing from fitted data distributions. (**A**) Frequency distribution based on simulated results using variation in mean density. (**B**) Number of pupae resulting from simulations that vary density across natural values. Circles are median, upper triangles are 75th percentile and down triangles are 25th percentile.

Using the total area of the study site and data from the fitted distributions of *Viola pur. quercetorum* density, number of leaves per plant, leaf area per leaf, and leaf area consumed, resulted in an asymmetric distribution (Figure 4A) with the average number of potential pupae on Chews Ridge being 765. The median number of pupae supported was 478, with 25% of the distribution being below 237 and 95% of the distribution being below 2403 pupae. In comparison the 2013 MRR estimate [24] was 227 butterflies (95% CI 146 to 392). The MRR estimate is at 23.7% of the simulated distribution, with lower and upper 95% CI at 12.9% and 42.3% respectively. The simulation varying density showed a strong linear relationship of increased number of pupae with increasing density (r^2^ = 0.99, *p* < 0.00001, y = 12,759.7x − 0.103, Figure 4B). As density increases, the variation in predicted number of pupae widens (Figure 4B) as a result of multiplying larger values of density by the other variables.

## 4. Discussion

The question of whether *S. adiaste* is limited by larval resources ultimately involves asking how many adults there *should* be in a given site. This paper presents the first dataset we are aware of quantifying the number of butterflies supported by the biomass of a *Viola* population. Our studies indicate there were 227 adult butterflies in 2013 and although the amount of plant *present* appears adequate to support this number, the amount of host *available* to larvae was much more limited because of plant distribution and associated larval movement. At face value the *Viola* abundance and biomass in 2013 predicted more pupae in 50% of simulation runs, so why were there “only” 227 butterflies in 2013? Herbivores do not generally eat all their host so should we expect all the host to be used? We discuss these questions along with several assumptions of our analyses below, both with respect to *S. adiaste*, and also to *Speyeria* in general.

Our results describing variation in plant distribution, abundance, density and leaf area present for its herbivore *Speyeria adiaste clemencei*, enabled us to predict how many individuals this site could sustain. The resulting asymmetric distribution of number of *S. adiaste* pupae strongly overlapped with the estimate and confidence interval for adult population size (Figure 4A), with the adult population size at approximately the 25th percentile of the distribution. However, given the asymmetric shape of the distribution of pupae, the 50th percentile was not far from the upper 95% CI of adult population size (392 adults vs. 478 pupae). Varying density had a strong effect on predicted number of pupae (Figure 4), with a positive linear relationship, similar to the results of Bierzychudek and Warner [31]. In our simulations it appears that only when relatively high values of plants per m^2^, number of leaves and leaf area are combined with relatively low values of leaf area consumed do the results deviate strongly from the observed number of adults (see Figure 4A,B). For example if the density were twice our observed value the median number of pupae predicted was 944 (Figure 4B). Thus our analysis predicts that a few hundred, and certainly less than a thousand *S. adiaste* individuals, could be supported by the *Viola* biomass present in this study, but we should not expect a *Viola* population such as this to support more than a thousand, and definitely not thousands, of adult *S. adiaste*.

Although our analysis predicts more pupae than observed adults, it is probably premature to say larval host is not limiting given several assumptions made that could be inflating our predicted values. Larvae were fed in small cups under lab, not field conditions. Thus our leaf consumption estimates may be lower than for free ranging larvae under fluctuating, and colder, field temperatures. In addition, using the native host could increase consumption estimates if larvae need to consume more non-native host to overcome different levels of chemical defense or extract sufficient nutrients. However, using the non-native host could decrease estimates if the native host is more defended or of lesser nutritive value. The fact that larvae of *Speyeria* species often feed on multiple hosts [8,38,39,40,41] and can be reared on non-native hosts including commercial pansies [23,38,41] makes it difficult to know how problematic this is and it should certainly be investigated further.

Additional assumptions regarding larval movement emphasize how the difference between amount of host *present* and amount *available* impacts estimates of population size. Our simulation analysis assumed that larvae find and consume every plant. This is not likely given the poor host finding ability documented in other *Speyeria* [30]. Furthermore, finding every plant assumes unlimited larval movement. In effect, host finding limitations decrease the amount of host *available* to larvae, making it much less likely that larvae find small, distant patches of host. For example, assuming the average transect density of 0.037 *Viola* per m^2^ and uniform density, together with average values for number of leaves per plant (=10.3) and average leaf area (=1.99 cm^2^), there is 0.76 cm^2^ food per m^2^ and based on our consumption estimates an average male would need 260 m^2^ of area to reach pupation, and a female 384 m^2^. These large areas highlight how potentially unavailable the host present may actually be.

In addition to *Viola* abundance, biomass and density, there are other aspects of *Viola* ecology that may be involved in “bottom–up” interactions that help explain the discrepancy between predicted pupae and adult estimates here, and declines in *Speyeria* in general. Host distribution is a potentially very important variable that can affect larval mortality. For example, the simulation study of Bierzychudek and Warner [31] showed that in addition to density, spatial distribution influenced pupation probability, and host finding success improved when *Viola* were arranged as randomly spaced clumps. In our study, *Viola purpurea quercetorum* on Chews ridge are not in dense carpets like some other *Viola* (e.g., *V. pedunculata*), but are heterogeneously distributed, with the densest patches widely separated (Figure 2). If we account for host distribution in our estimation of total number of pupae by estimating the number of transect segments 30 m in length that have enough plants to support a pupa (10 or more based on our estimated consumption rates if we assume average leaf area), there are 34 such transect segments (22,260 m^2^ sampled). Since the area sampled represents 9.2% of the total study area, then a naïve extrapolation would yield 371 supported pupae. This value is within the 95% CI of estimated number of adults in 2013.

It may also be important to account for competition that results from the clumped distribution. If females preferentially lay eggs near appropriately sized clumps of host, rather than seeking isolated plants, it follows that many larvae are present in a clump. Although we did not observe larval feeding behavior in the field, it seems reasonable that *S. adiaste* are similar to other *Speyeria* and stay near a plant they are consuming [31], and are unable to locate hosts from more than a few centimeters away [30]. Assuming these aspects of *S. adiaste* larval ecology are true, the implication is that larvae may need to leave a clump as a result of scramble competition. This exposes larvae to starvation as they seek additional hosts and reduces the number of eclosing adults compared to amount of host present.

Finally, induced defenses in *Viola* hosts could make the host biomass *present* unequal to the host biomass *available* or useable by larvae. Even if a host is easily found by a larva, some leaves or individuals may be poor quality or inedible because of previous herbivory [42]. A study with spider mites and pansies (*Viola* × *wittrockiana* Gams) showed that inducing plant defenses with methyl jasmonate caused the herbivores to disperse more quickly from treated plants [43]. Several *Viola* species are documented to have cyclotides [44,45,46,47,48,49], proteins which have been shown to provide defense against lepidopterans [50,51,52] and to have antimicrobial activity [46]. If these defenses are generally applicable, *Viola* that have been eaten by *Speyeria* may have higher concentrations of herbivore defenses that make the individual leaf or plant less palatable or even deadly. Observations in the field seem consistent with this, in that during the surveys for this project we observed only part of a leaf or one leaf and not an entire plant eaten by *Speyeria adiaste* (RIH and CER personal observation). This could indicate larvae eat one leaf and move on, but field observations are needed to confirm this since the pattern may result from smaller larvae sheltering nearby and returning over and over to eat.

The differences we observed in male and female consumption have implications for female fecundity, sex ratio and how many adults can be sustained. Males consumed significantly less than females, and males were significantly smaller than females in both the lab and field samples. For males the small size and lower leaf consumption may relate to selection for protandry—males eclosing earlier have access to the first females eclosing. However, this may be counteracted by male-male competition where larger males or males with larger thoraxes win competitive bouts for hilltops. For females, the association of higher leaf consumption and larger size should relate strongly to fecundity, as fecundity is affected by food limitation in *Speyeria mormonia* [53], and is positively correlated with adult size among butterfly species [54]. However, no data on the relationship between female fecundity and body size or leaf consumption are available for *S. adiaste*, and few other *Speyeria* have been studied. *Speyeria idalia* females are smaller where host density is lower [8], and in *S. mormonia* food limitation affects number of eggs [53], but body size does not [53,55], although very large females lay larger eggs [55]. If food limited or smaller females do lay fewer eggs (or smaller eggs) then this represents a food limitation mechanism for population declines other than larval mortality. In addition, the number of males that can be sustained is larger than the number of females given that females consume more host leaf. Furthermore, if plant density or spatial arrangement are suboptimal, this may have a disproportionate effect on female mortality, reducing female numbers and contributing to male biased adult sex ratios and population declines.

Although food resources are obviously important for herbivores and have been shown to be important in this study for *S. adiaste*, top–down interactions from predators and parasites may also be important. Little is known about the parasites of eggs, larvae and pupae of *Speyeria*, but *Madremyia* tachinid flies are likely important larval parasitoids given their wide Lepidoptera host range, and documentation on multiple *Speyeria* [56,57]. Predators likely play a significant role in larval mortality in *S. adiaste* and *Speyeria* in general. For example, Bierzychudek and Warner [31] observed predation attempts by ants and folding-door spiders (*Antrodiaetus* sp.) on *S. zerene hippolyta* searching for host plants in the field. Bierzychudek and Warner [31] stated that predation risks increase with more time looking for host plants. This predation risk would be higher in lower densities or spatial arrangements requiring larvae to travel farther. Predation would also likely affect female larvae disproportionately and in turn the adult sex ratio, given that females require more host plant and presumably move more to find it. Learning more about parasitoids and predators of *S. adiaste* would be worthwhile and given the difficulty finding larvae in the field [23] methods similar to Bierzychudek and Warner [31] using lab reared larvae could provide useful data on predation and possibly parasitism rates.

Our approach provided a detailed snap shot of a single *Viola purpurea quercetorum* population at a local scale, but like most methods it has benefits and limitations. Limiting assumptions have been discussed above, but a benefit of this local scale approach is that it provided more detailed data on host plant ecology and larval food limitation compared with correlations of host abundance among many populations. As such, the distributions and summary statistics of plant traits and larval consumption can be useful in calculating target population sizes of *Viola* or *Speyeria*. The approach taken here is also useful for depicting variation in the system by incorporating stochastic rather than deterministic values for different variables. For example, drawing from the distribution of leaf area consumed that included both males and females included a degree of realism given that leaf area consumed will depend on larvae finding the hosts and our data were for larvae fed *ad libitum*. With more data on leaf consumption it would be possible to simulate males and females separately, but they share the resource so assumptions about sex ratio would need to be made.

As mentioned in the above paragraphs, there are many opportunities for further research in this system. One important area is to obtain data on leaf consumption on native larval hosts to investigate variation on different native hosts, as well as to measure size of resulting adults and female fecundity. It would also be interesting to investigate larval behavior consuming plants that have or have not been partly eaten by larvae. The excess host observed in our study is a good thing because it indicates larval resources are present to support more butterflies in some years—but temporal variation needs to be investigated. Replicating this study at a smaller scale within the Chews Ridge study area across years would help clarify the food limitation hypothesis. For example, tracking abundance in the densest patches and using the distributions for other plant traits could demonstrate a correlation with adult counts, as well as any discrepancies between predicted number of pupae and adult counts. Studying *Viola* and *Speyeria* at additional sites could also provide valuable data on natural *Viola* spatial distribution and density to know whether a site could support more butterflies given the host present.

## 5. Conclusions

Our results strongly corroborate and complement research on host–butterfly interactions in other *Speyeria* species providing useful data for management and restoration. Monte Carlo simulations indicated that the adult population estimate fell in the first quartile of what can be expected based on host density and biomass *present* at the site. Although these results suggest at face value that the *S. adiaste* population was not food limited, they should be interpreted carefully because the analysis included several assumptions that would lower the actual number of butterflies supported. Overall it would be reasonable to expect a few hundred, but certainly not more than one thousand adult *S. adiaste* based on host resources *present* in the study site. Although more information is needed on larval behavior of *S. adiaste*, and other *Speyeria*, and on how different *Viola* are distributed, results of this study and other recent work provide useful guidelines and tools for management and restoration of *Viola* food plants. For example, the models here can be used to understand whether larvae in a population appear food limited by calculating the number of adults that could be theoretically sustained by the host density present. Conversely, they can also be used in calculating the density of host required for a target number of adults. Even with the assumptions of our study, the amount of “excess” host observed in our analysis indicates that to achieve a robust population for a target number of butterflies, the *Viola* density should be twice or more what is required based on mere biomass. In addition to ensuring density is more than adequate, randomly spaced clusters of plants will likely increase the probability of encountering another plant before larvae starve or are predated [31]. Management and restoration of *S. adiaste*, and other *Speyeria*, should focus on not only maintaining excess levels of density, but also on the spatial distribution of larval food plants.

## Appendix A

Truncated normal distribution R code.

We called it dtnorm2 to differentiate between the packages dtnorm():

dtnorm2<- function(x, mean, sd, a, b) {
dnorm(x, mean, sd)/(pnorm(b, mean, sd)-pnorm(a, mean, sd))
}
ptnorm <- function(x, mean, sd, a, b) {
(pnorm(x,mean,sd) - pnorm(a,mean,sd)) /
 (pnorm(b,mean,sd) - pnorm(a,mean,sd))
}

## Appendix B

Results of model fitting for the number of leaves per plant, leaf area per leaf, and leaf area consumed datasets.

**Table A1:** Number of leaves per plant (*V. purpurea*).

Distribution	Likelihood	AIC
log-normal	−778.7	1561.5
gamma	−780.2	1564.4
weibull	−784.2	1572.3
trunc-normal	−793.5	1590.9
normal	−841.0	1686.0

**Table A2:** Leaf area per leaf (*V. purpurea*).

Distribution	Likelihood	AIC
gamma	−2107.8	4219.5
weibull	−2108.5	4221.0
trunc-normal	−2119.5	4242.9
log-normal	−2122.9	4249.7
normal	−2138.3	4280.6

**Table A3:** Leaf area *V. papilionacea* consumed.

Distribution	Likelihood	AIC
weibull	39.3	−74.6
normal	38.7	−73.4
trunc-normal	38.7	−73.4
gamma	38.3	−72.6
log-normal	38.0	−72.0
